# Malformations Caused by Shuni Virus in Ruminants, Israel, 2014–2015

**DOI:** 10.3201/eid2112.150804

**Published:** 2015-12

**Authors:** Natalia Golender, Jacob Brenner, Motti Valdman, Yevgeny Khinich, Velizar Bumbarov, Alexander Panshin, Nir Edery, Shimon Pismanik, Adi Behar

**Affiliations:** Kimron Veterinary Institute, Bet Dagan, Israel (N. Golender, J. Brenner, Y. Khinich, V. Bumbarov, A. Panshin, N. Edery, A. Behar);; Hachaklait, Caesarea, Israel (M. Valdman);; Israeli Veterinary Field Services, Gilboa, Israel (S. Pismsanik)

**Keywords:** Shuni virus, Simbu virus, orthobunyaviruses, viruses, Israel, malformation, congenital abnormalities, emerging disease, ruminants, animals

**To the Editor**: Viruses in the Simbu serogroup are arboviruses that cause abortion, stillbirth, and congenital abnormalities in domestic ruminants. Akabane virus (AKAV), Aino virus (AINV), and Schmallenberg virus are the most studied in this serogroup; Shuni, Sabo, Shamonda, and Sango viruses (*1*,*2*) are examined less frequently. Until 2012, only AKAV had been associated with congenital abnormalities in Israel, although AINV had been identified serologically in dairy cow herds with no clinical signs in 2003 (*3*). Moreover, of 15 brain samples collected during February–October 2012 from adult cows with central nervous system manifestations, 6 were positive for AKAV by PCR.

In late December 2014, the Israeli Veterinary Field Services was notified of the appearance of arthrogryposis-hydranencephaly syndrome (*1*) in 2 herds of sheep in the villages of Yokneam and Sde Ya’akov, respectively; both villages are located in the Izre’el Valley, in Israel’s northern valleys (Technical Appendix Figure 1), where several arboviral infections have occurred in recent decades. From our past experience (*3*), ≥1 virus of the Simbu serogroup was suspected to have infected the ruminants, probably during August–October 2014.

We collected 27 samples of brain, placenta, spleen, lung, and blood (mixed with EDTA to prevent coagulation) from 15 sheep, goats, and cattle. Most samples were from the 2 affected flocks in the northern valley; a few were from ruminants in additional locations: Avadon, near Israel’s border with Lebanon; Ein Hachoresh, near central Israel; and Hura, close to the Negev desert (Technical Appendix Figure 1). 

Of the 27 samples, 23 (85%) were positive for Shuni virus (SHUV) by PCR (Table). SHUV, which had not been reported in Israel, was isolated from the brain and placenta of 1 malformed lamb (strain 2504/3/14; sample 11 in the Table). Moreover, partial nucleotide sequences of the small, medium, and large DNA segments (580/850, 4,320/4,326, and 285/6,880 bp, respectively) were identified from 3 samples (strains Yokneam 2417/2/14 and 2504/3/14 and Hura 273/14 from samples 2, 11, and 9, respectively, in the Table; Technical Appendix Figure 2). Sequence data obtained by conventional PCR in this study have been deposited into GenBank (accession nos. KP900863–5, KP900873–5, KP900879–80, and KP900884). Phylogenetic analysis of the samples showed that they were isolates of SHUV (Technical Appendix Figure 2). Additional SHUV RNA-specific fragments were detected in pathologic samples from kids, lambs, and calves (Table). Full-genome sequences were not performed, although sequencing should be done when possible to determine precise origin of isolates.

**Table:** Summary of diagnostic and laboratory findings, animal species, sample materials, and region where samples were collected in the study of Shuni virus infection in ruminants, Israel, 2014–15*

Animal no.	Laboratory no.	Species	Clinical manifestation	Region	PCR-positive sample	Vero isolation	Virus isolation in mice
1	2417/1/14	Sheep	Malformed, aborted fetus	Northern valley	Brain, placenta	Negative	Negative
2	2417/2/14	Sheep	Malformed, aborted fetus	Northern valley	Brain	Negative	Negative
3	267/2/14	Sheep	Malformed, aborted fetus	Northern valley	Brain	Not done	Not done
4	267/3/14	Sheep	Malformed, aborted fetus	Northern valley	Brain	Not done	Not done
5	267/4/14	Sheep	Malformed, aborted fetus	Northern valley	Brain	Not done	Not done
6	2498/1/14	Sheep	Weak lamb syndrome	Northern valley	Brain, EDTA-blood	Negative	Negative
7	2504/1/14	Sheep	Malformed aborted fetus	Northern valley	Brain	Not done	Not done
8	2504/2/14	Sheep	Malformed, aborted fetus	Northern valley	Brain, placenta	Negative	Negative
9	273/14	Sheep†	Malformed, aborted fetus	Negev	Brain	Not done	Not done
10	274/14	Sheep	Aborted fetus	Northern valley	Brain, placenta	Not done	Not done
11	2504/3/14	Sheep†	Malformed, aborted fetus	Northern valley	Brain, placenta	Positive	Positive
12	275/1/14	Sheep	Malformed, aborted fetus	Northern valley	Brain, placenta	Negative	Negative
13	275/2/14	Sheep	Malformed aborted fetus	Northern valley	Brain, placenta	Not done	Not done
14	263/14	Goat	Malformed, aborted fetus	Northern valley	Brain, placenta	Not done	Not done
15	215/14	Cattle	Aborted fetus	Upper Galilee	Brain	Negative	Negative

*Not done, not performed if insufficient brain material was available for cerebral inoculation or if the infected brain failed to propagate in the cell line. For some animals, >1 sample was collected. †Sequences used to build the phylogenetic trees in Technical Appendix Figure 2.

For further testing, we inoculated homogenate material from 7 distinct malformations (samples 1, 2, 6, 8, 11, 12, and 15 in the Table) into baby mice; only 1 family of baby mice inoculated intracerebrally with the SHUV isolate (sample 11 in the Table) exhibited characteristic neurologic signs of nervousness. PCR confirmed that SHUV caused the cerebral infections in these mice. The isolate was also suitable for further propagation in the Vero cell line (Table).

Our results showed the presence of SHUV in sheep in Israel during the winter of 2014–15 and suggest a northward expansion of SHUV from sub-Saharan Africa. Although SHUV was first isolated in the 1960s (*2*), its role as a pathogen has been shown only recently in its involvement in encephalitis in horses (*4*). We isolated SHUV from the pathologic fetal brain of a malformed lamb, an unusual laboratory finding because, although Simbu viruses are readily isolable from vectors or exposed animals during the 3 or 4 days of viremia, they are seldom isolable from pathologic specimens collected for study of congenital malformations. We deduce from the clinical evidence that malformations appear up to 6 months after infection with SHUV and after the virus has been eliminated from the host after immune activity. Thus, isolation of SHUV from malformed brains may indicate strong neurotropism of this putative pathogen. The possibility of its replication in the fetal nervous system should also be considered because an affected fetus that is born alive is likely a reservoir. Indeed, AKAV was identified in the hippocampus (only) of adult lactating cows (data not shown), and similar epidemiologic evidence might result from other Simbu virus infections.

A serologic survey conducted in Israel during the 2001–2003 outbreaks of AHS showed reactivity of AINV to serum samples of ruminants in Israel’s southern regions (*3*). Because AINV and SHUV are known to have a strong serologic cross-reaction, SHUV has likely previously infiltrated Israel. However, whether the seroreactivity results from AINV or SHUV remains unresolved.

The emergence and reemergence of arboviruses should interest medical practitioners, particularly epidemiologists. The appearance of exotic viruses in unexpected locations might result in more severe pathology in newly invaded regions than in the original arbovirus-endemic areas. Furthermore, SHUV has been detected in a child with febrile illness (*2*), a finding that suggests a potential zoonotic problem.

**Technical Appendix.** Map showing locations where Shuni viruses were detected in ruminants in Israel, 2014–15, and phylogenetic trees of Simbu serogroup viruses.
