# Supplementary material for: Malformations Caused by Shuni Virus in Ruminants, Israel, 2014–2015
Source: Emerg Infect Dis. 2015 Dec;21(12):2267–8. doi: 10.3201/eid2112.150804 (PMC4672418; doi:10.3201/eid2112.150804)
Supplement: Supplementary file 1 — Technical Appendix. Map showing locations where Shuni viruses were detected in ruminants in Israel, 2014–15, and phylogenetic trees of Simbu serogroup viruses. [file 15-0804-Techapp-s1.pdf]

Article DOI: http://dx.doi.org/10.3201/eid2112.150804

Malformations Caused by Shuni Virus in Ruminants, Israel, 2014–15

Technical Appendix

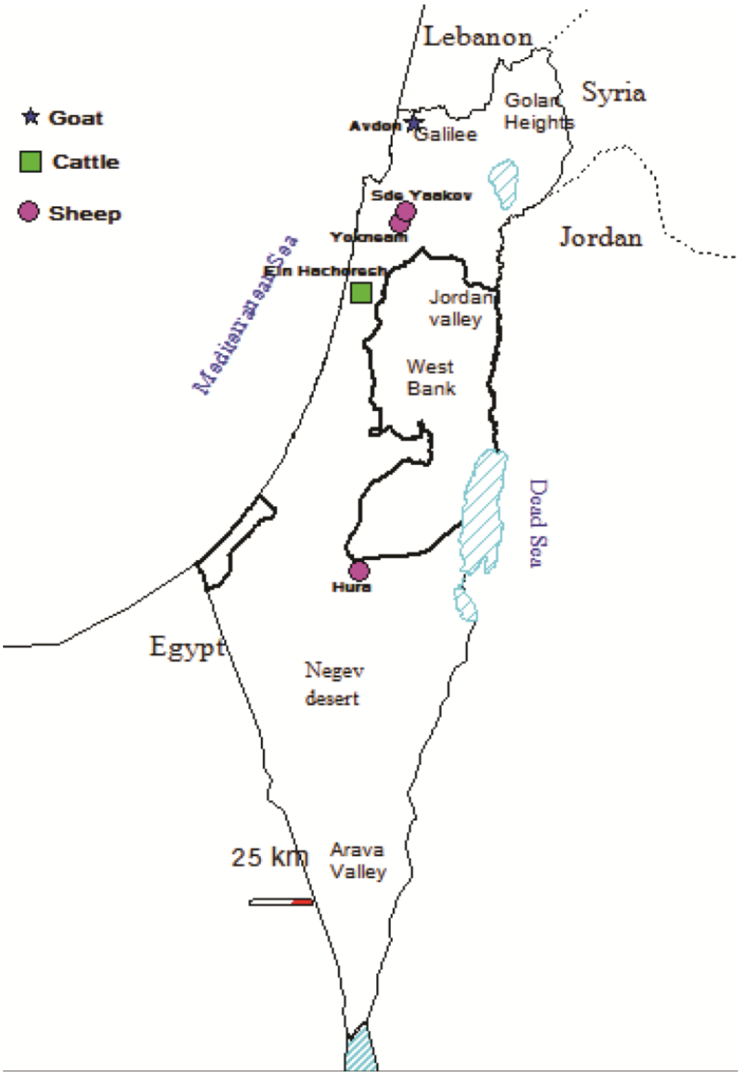

Technical Appendix Figure 1. Map showing locations where Shuni viruses were detected in ruminants in Israel, 2014–15. Scale bar indicates distance.

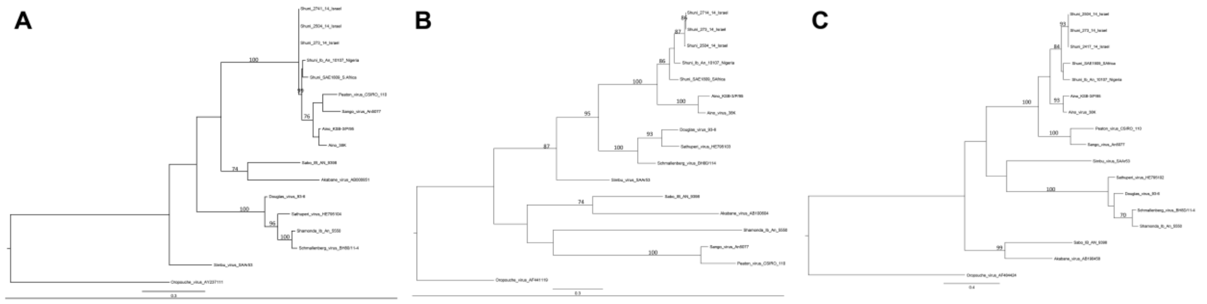

Technical Appendix Figure 2. Rooted maximum-likelihood phylogenetic trees of Simbu serogroup viruses. Segments were constructed on the basis of a general time-reversible and gamma-distributed rate heterogeneity (GTR_G) model of nucleotide substitution. A) S segment; B) M segment; and C) L segment. Phylogenetic analysis was performed by using PhyML (*1*,*2*). Values above the branches indicate bootstrap support. Only bootstrap values >70% are shown. The segments of the Israeli isolates were compared with the appropriate sequences from validated Simbu viruses. When possible, we used Simbu serogroup viruses with available full-segment sequences that have been validated according to Goller et al. (*3*) and van Eeden et al. (*4,5*). For additional Aino viruses for which full-segment sequences were not available, we used GenBank accession numbers from previous studies (*5,6*). Homologous sequences from Oropouche virus were used as an out-group. Scale bar indicates estimated nucleotide substitutions.

References

1. Dereeper A, Guignon V, Blanc G, Audic S, Buffet S, Chevenet F, et al. Phylogeny.fr: robust phylogenetic analysis for the non-specialist. Nucleic Acids Res. 2008;36:W465–9. http://dx.doi.org/10.1093/nar/gkn180

2. Guindon S, Gascuel O. A simple, fast, and accurate algorithm to estimate large phylogenies by maximum likelihood. Syst Biol. 2003;52:696–704. http://dx.doi.org/10.1080/10635150390235520

3. Goller KV, Höper D, Schirrmeier H, Mettenleiter TC, Beer M. Schmallenberg virus as possible ancestor of Shamonda virus. Emerg Infect Dis. 2012;18:1644–6. http://dx.doi.org/10.3201/eid1810.120835

4. van Eeden C, Williams JH, Gerdes TG, van Wilpe E, Viljoen A, Swanepoel R, et al. Shuni virus as cause of neurological disease in horses. Emerg Infect Dis. 2012;18:318–21. http://dx.doi.org/10.3201/eid1802.111403

5. van Eeden C, Harders F, Kortekass J, Bosser A, Venter M. Genomic and phylogenetic characterization of Shuni virus. Arch Virol. 2014;159:2883–92. http://dx.doi.org/10.1007/s00705-014-2131-2

6. Yanase T, Aizawa M, Kato T, Yamakawa M, Shirafuji H, Tsuda T. Genetic characterization of Aino and Peaton virus field isolates reveals a genetic re-assortment between these viruses in nature. Virus Res. 2010;153:1–7. http://dx.doi.org/10.1016/j.virusres.2010.06.020
